# Monkey Bar Dimensions Associated with Pediatric Upper Extremity Fractures Show Deviations from United States Product Safety Commission Recommendations

**DOI:** 10.7759/cureus.6534

**Published:** 2020-01-01

**Authors:** Marissa P Teitelbaum, Lawrence Stankovits, Evan Curatolo

**Affiliations:** 1 Engineering, High Technology High School, Lincroft, USA; 2 Pediatric Orthopedics, RWJBarnabas Health, Long Branch, USA; 3 Pediatric Orthopedics, Atlantic Pediatric Orthopedics, Shrewsbury, USA

**Keywords:** jungle gym, playground, horizontal overhead ladders, fracture, trauma

## Abstract

Background

Monkey bar injuries account for the majority of playground injuries, and 34% result in a fracture. Studies have shown that there has been no decline in the number of monkey bar injuries over several decades. Our goal was to focus on fractures of the upper extremity resulting from monkey bar injuries. Additionally, we set out to analyze the dimensions of the monkey bar apparatus on which the injury occurred and determine if they were compliant with those recommended by the United States (US) Product Safety Commission.

Methods

A retrospective chart review of all upper extremity injuries seen in a large pediatric orthopedic practice in 2017 was conducted to find all monkey bar-related injuries. Data was collected including age at the time of injury, gender, and injury type. Families of the injured child were contacted to identify the exact location of the injury. On-site measurements were made of the monkey bar apparatus including height, the distance between grips, the circumference of the grip, and ground surface type.

Results

Of 1968 patients seen in 2017, there were 990 upper extremity injuries and 66 monkey bar injuries (98.5% fractures). The average age of those injured on monkey bars was 6.6 ± 1.9 years, 60.6% were males. The average height of the apparatus was 207.32 ± 16.59 cm (range: 176.53-254 cm), the average distance between grips was 35.30 ± 5.62 cm, and the average circumference of the grip was 9.83 ± 1.03 cm. All exceeded the recommended height for preschool children aged 4-5 years (152.4 cm), and 11 of the 30 (36.7%) exceeded this recommended height for school-age children (213.26 cm). For the distance between grips, 23 of the 30 (76.7%) exceeded the preschool recommendation (30.48 cm), and three of 30 (13.3%) monkey bars exceeded the recommendation for school age children (38.1 cm).

Conclusion

Monkey bar injuries continue to be a common source of upper extremity fractures among young children. There is a high rate of non-compliance with current recommended safety standards.

## Introduction

According to the Consumer Product Safety Commission (CPSC) of the United States of America, which includes data from the National Electronic Injury Surveillance System (NEISS), there were 1,459,201 playground injuries treated in emergency departments (ED) from 2009 to 2014 [1]. Among the total number of injuries, 61% occurred at schools or parks and 14% at home. Of those injuries seen in the ED, 499,797 (34%) occurred on monkey bars and 34% of the injuries resulted in a fracture. A study by Loder analyzing playground equipment injuries from 1991 to 2005 using NEISS data revealed that monkey bar injuries were the most common cause of fractures [2]. They specifically noted that the number of injuries per year per capita owing to monkey bars was stable as compared to declining rates for swings and slides. This study, as well as a 2006 to 2014 analysis by Tuckel et al., conclude that continued efforts are needed to ensure that playgrounds constitute a safe environment for childhood recreation [3]. Accordingly, the Public Playground Safety Handbook published by the United States (US) Product Safety Commission provides suggestions as to the appropriate dimensions of monkey bar apparatuses, as well as space requirements and fall surface characteristics [4].

Despite the high morbidity, overall few studies have focused specifically on monkey bar injuries. We, therefore, set out to analyze significant monkey bar-related injuries by analyzing those seen within an orthopedic practice, thus focusing more on fractures. We suspect that the injuries obtained were more related to the height of the fall from the equipment and thus sought to analyze the various dimensions of the monkey bar apparatus where the injuries occurred and compare them to the national recommendations.

## Materials and methods

The authors performed a retrospective chart review of children (age: from birth to 20 years) seen in the office of a private two-physician pediatric orthopedic surgery practice in the year 2017. According to the estimated census data from the US Census Bureau, the region covered by this practice includes a suburban population in New Jersey of roughly 240,000 children [5].

Upper extremity injuries are more common than lower extremity injuries when monkey bars are involved. Therefore, we focused our data collection on upper extremity injuries, including fractures and sprains. An electronic billing database was used to search all upper extremity injuries occurring in 2017 using ICD 10 codes S40 to S69. The identified patients’ paper charts were then reviewed and data concerning the age of patients at the time of injury, gender, type of injury, and mechanism of injury were recorded. The mechanism of injury was categorized into larger groups including monkey bars, playground, bicycle, falls, sports, trampoline, punching, other, and unknown. The playground category includes those injuries occurring on a playground, unless they specifically noted that the injury occurred on the monkey bars.

To ensure the accuracy of the electronic database in identifying all upper extremity injuries, a convenience sample of all charts representing those patients seen in 2017 with last names A through G was reviewed as well. The total number of patients seen in 2017 was also recorded. For those patients with monkey bar or playground-related injuries, efforts were made via phone call and email to determine the location of the injury.

An attempt was made to visit the monkey bars on which accidents occurred. For each, the height of the monkey bar apparatus was obtained, from the top of the grip to the ground below. In areas where the ground surface was uneven or the bars were at various heights, the measurement was made where the greatest distance existed. The circumference of the grip, as well as the distance between grips, was recorded. Notation was also made as to the shape of the grip (e.g. straight bar, circle, teardrop), the trajectory of the course/travel pattern (e.g. straight, “s” shaped) as well as the type of surface material underneath. Measurements were obtained from a commercially produced backyard monkey bar set of one author as representative of “home” monkey bars, although no injuries had occurred on them. The surface underneath these monkey bars was not included in the analysis as it would be different in different homes.

## Results

A total of 990 patients were identified with upper extremity injuries, of these 61.0% were male. The average age was 9.6 ± 4.1 years (range birth to 20 years). Review of the convenience sample of patients with last names A through G identified 646 patients, of which 436 had upper extremity injuries. All of these patients were included in the electronic database used to identify upper extremity injuries. A total of 1968 children were seen in 2017.

In analyzing the mechanism of injury, there were 66 monkey bar-related injuries, occurring with a male predominance (60.6%). The average age was 6.6 ± 1.9 years (range: 3-11 years). Playground injuries occurred in 61 children, 45.9% males, with an average age of 6.9 ± 2.9 years (range: 2-14 years). The remaining distribution of age and gender based on the mechanism can be found in Table 1.

**Table 1:** Mechanism of injuries

Mechanism	n	Male n (%)	Avg. Age (yrs)	Std. Dev.	Range
Bicycle	39	32 (82.1)	10.1	2.3	3–15
Fall / Collision	298	171 (57.4)	7.7	3.9	5 months to 20 years
Monkey Bars	66	40 (60.6)	6.6	1.9	3–11
Other Accident	139	76 (54.7)	9.2	4.1	Birth to 17
Playground	61	28 (45.9)	6.9	2.9	2–14
Punch / Fight	14	13 (92.9)	14.7	1.9	11–17
Sport	303	196 (64.7)	12.0	2.9	4–18
Trampoline	25	13 (52.0)	7.7	3.6	2–17
Unknown	45	31 (68.9)	9.3	3.9	10 months to 16 years
Total	990	604 (61.0)	9.6	4.1	Birth to 20 years

The most common injury recorded from the monkey bars was a fracture of the distal radius in 37 patients, of which 17 also had a fracture of the distal ulna. The second most common injury was a supracondylar fracture in 17 children. Among those injured on the playground, 20 had a fracture of the distal radius, with seven also fracturing the ulna, as well as 20 with a supracondylar fracture. The types of injuries are further delineated in Table 2.

**Table 2:** Type of injury

Type of Injury	Monkey Bars n (%)	Playground n (%)	All Others n (%)
Clavicle Fracture	1 (1.5)	1 (1.6)	51 (5.9)
Distal Humerus Fracture	7 (10.6)	6 (4.9)	32 (3.7)
Distal Radius Fracture	20 (30.3)	13 (21.3)	306 (35.6)
Distal Radius and Ulna Fracture	17 (25.8)	7 (11.5)	86 (10.0)
Elbow Sprain	0 (0)	1 (1.6)	19 (2.2)
Metacarpal Fracture	0 (0)	1 (1.6)	41 (4.8)
Phalanges Fracture	0 (0)	1 (1.6)	66 (7.7)
Proximal Radius Fracture	1 (1.5)	8 (13.1)	23 (2.7)
Proximal Ulna Fracture	2 (3.0)	3 (4.9)	15 (1.7)
Supracondylar Fracture	17 (25.8)	20 (32.8)	73 (8.5)
Wrist Sprain	1 (1.5)	0 (0)	18 (2.1)
Other Injuries	0 (0)	0 (0)	133 (15.5)

A total of 49 monkey bars or playground sites where the injury occurred were identified. Three of these sites had two individuals each sustaining injuries at that location during the study period. These three pieces of equipment are thus “counted twice” as two separate individuals sustained injuries on them. Among the total, 14 playgrounds were inaccessible (private home (six), locked facility (seven), and too distant (one)). The remaining 35 sites were available to be measured, of which six of the playgrounds did not contain monkey bars.

The average height of the apparatus, distance between grips, and circumference of the grip can be found in Table 3. This data has been divided based on the location of the monkey bars into groups including elementary schools, kindergarten through eighth grade, middle school, and parks as well as total values. The representative home monkey bars were level and straight with a straight bar and was 281.44 cm in height, with a distance between grips of 27.94 cm, and a grip circumference of 10.80 cm.

**Table 3:** Measured dimensions of the monkey bar apparatus

	Height (cm)	Distance (cm)	Circumference (cm)
Elementary School (n = 20)			
Average	209.1	34.3	9.9
Standard Deviation	17.7	5.1	1.1
Range	182.9–254	27.9–49.5	8.9–11.4
K-8 (n = 3)			
Average	211.7	42.8	9.5
Standard Deviation	2.9	9.5	1.1
Range	208.3–213.4	31.8–48.3	8.9–10.8
Middle School (n = 2)			
Average	215.3	36.2	9.8
Standard Deviation	0.9	0.9	0.4
Range	214.6–215.9	35.6–36.8	9.5–10.2
Park n=4			
Average	191.1	34.3	9.8
Standard Deviation	11.8	1.8	1.1
Range	176.5–204.5	33.0–36.8	8.9–10.8
Total (n = 29)			
Average	207.32	35.30	9.83
Standard Deviation	16.59	5.62	1.03
Range	176.53–254	27.94–49.53	8.89–11.43

The surface underneath the apparatus was mulch (21), foam (four), and grass/dirt (four). The design of the monkey bar apparatus was quite variable. The grip was identified as a straight bar (eight) of teardrop shape with a flat bottom (13) with one of them allowing for front-back movement and one at the end of a chain; circular ring (four) one allowing movement both front-back and side to side, another only front-back movement, the top half of a semi-circle; S-shaped; and irregular shaped (two). As to the trajectory of the course/travel pattern, there was also variability with designs including level and straight (12); arched and straight (two); level in a circular path (four); level in a C-shape (seven); level S-shaped (one); Z-shaped (one); and irregular (two).

## Discussion

Our data suggest that monkey bars continue to be a major cause of injury to children. Evaluation of the NEISS data for monkey bar injuries from 2008 to 2017 reveals no evidence of a decline (79,020 injuries in 2008 and 79,023 injuries in 2017, with similar data points in between ranging from 78,024 to 94,219) [1]. This is similar to the lack of decline described by Loder from 1991 to 2005 [2]. Similarly, in 2005, there were 80,312 monkey bar injuries [1]. The 66 upper extremity monkey bar injuries in our study accounted for 6.7% of all upper extremity injuries seen within the orthopedic group in 2017 and 3.4% of all children seen that year. As the designation of playground as the site of injury in an additional 61 children likely includes some additional monkey bar injuries, the maximal proportion of upper extremity or total injuries could be 12.8% and 6.5%, respectively.

To our knowledge, this is the first study to specifically look at injury rates requiring follow-up within the orthopedic specialty. It is worth noting that while the NEISS data looked at all ED visits for playground-related injuries, only 34% resulted in a fracture. A more recent study found that 64.2% of falls specifically from monkey bars resulted in fractures. [6] As our study acquired its data from follow-up visits to the orthopedics’ office, nearly all were visits were related to fracture since lacerations, contusions and most sprains did not require orthopedic follow-up. Indeed, among the 66 monkey bar injuries, 65 were fractures. Thus, these numbers are consistent with those of Waltzman who found 124 fractures related to monkey bar injuries over a two-year period in Children’s Hospital Boston, a large urban Children’s Hospital/Regional Pediatric Trauma Center [7].

The average age of the children sustaining monkey bar injuries of 6.6 years was also consistent with prior literature. One can see that within our own data reflecting upper extremity trauma, this group had the lowest average age (Table 1). Waltzman’s patients with monkey bar injuries similarly had an average age of six years, and Loder’s study using NEISS and other databases revealed an average age of 6.5 years with respect to playground-related injuries [2,7]. Similarly these studies, like ours, also showed a male predominance.

The types of injuries obtained on playgrounds were analyzed by Waltzman who found a high incidence of long bone and supracondylar fractures [7]. They described 17% with a radius fracture, 33% with a radius/ulna fracture, and 40% with a supracondylar fracture. This is similar to our patients with a proximal or distal radius fracture (21/67 [31.8%]), radius/ulna fracture [25.8%] and supracondylar fracture [25.8%]). It is notable that in their study, 7% suffered a lower extremity fracture. However, our study focused on upper extremity injuries as anecdotal observation and the pre-existing literature suggested they are far more common. Waltzman suggested that upper extremity injuries were more common as younger children’s balance was not fully developed [7]. Also, as their center of gravity is more cephalad, they tend to land on their upper extremities, torso, and head.

The Public Playground Safety Handbook published by the US Product Safety Commission addresses suggested guidelines for monkey bars (described as horizontal overhead ladders and overhead rings) [4]. Although they do not provide data for their recommendations, they suggest that for preschool (age: 2-5 years) equipment, the maximal fall height should be 60 inches (152.4 cm). In analyzing our data, 100% of the apparatuses, including the home monkey bars, exceeded this height. One can specifically see from our data that places where preschool children might play such as in the K-8 school and the parks, the average heights were quite high, 211.7 and 191.1 cm, respectively. The handbook also states for school-aged children (age: 5-12 years), the maximum fall height should be 84 inches (213.26 cm). In evaluating our data from parks and schools, as well as the representative home monkey bars, 11 of the 30 (36.7%) exceeded this recommended height. It is notable that there was a general trend in increasing average heights from parks where all age children play, to elementary schools, to K-8, to middle school. However, the tallest monkey bars, 254 cm, were at an elementary school.

A study by Chalmers et al. in 1996 studied playground injuries in New Zealand [8]. They analyzed 300 children age 14 or less who sustained a fall from playground equipment, of which 110 sustained an injury. They performed logistic regression analysis to reveal that falls from heights in excess of 150 cm increased the risk of injury 4.1 times that of falls from 150 cm or less. At the time, the maximal permissible fall height in New Zealand was 250 cm. Mott analyzed fall heights as they relate to fracture in the United Kingdom and similarly found a relation between fall height and fracture risk [9]. Of the 30 observed fractures in their study, only one occurred at a height 150 cm or less. A maximal height of 150 cm or less is particularly reasonable since the average height of 6.5-year-old boys and girls is 119 and 118 cm, respectively. Our study did not specifically address the question as to whether the increase in height of the apparatus above the recommendation is the cause of the fracture, although the previously cited studies suggest this is true [8-9]. Future prospective studies or studies comparing the rate of fractures occurring on monkey bars that adhere or exceed recommendations can be performed.

The handbook also provides suggestions for the maximal distance between grips. For preschool children, they suggest a maximal distance of 12 inches (30.48 cm), based on our data this was exceeded by 23 of the 30 (76.7%) monkey bars. For school-aged children, the recommended maximum was 15 inches (38.1 cm), which was exceeded by three of 30 (13.3%) of our studied monkey bars.

We also analyzed the circumference of the grip. While this had not been previously studied and the handbook provides no guidance, we noted only small deviations in this measurement. The average circumference of 9.83 cm seems appropriate given the average hand length of 6.5-year-olds is 13.2 ± 0.8 cm [10].

Studies evaluating whether the surface underneath playground equipment could decrease injuries have not proven such a relationship. Walzman attempted to address this question by surveying the families of injured children by telephone regarding the play surface [7]. They concluded that the injury pattern was independent of the surface type. Similarly, Sosin could find no difference in injury rates between impact-absorbing surfaces and grass [11]. Mott et al., however, demonstrated that mulch and rubberized surfaces were safer than concrete surfaces in preventing playground injuries [9]. However, mulch was not significantly more protective than concrete with respect to arm fractures. Choi examined peak vertical and horizontal force of impact after a fall utilizing various surfaces including mulch, rubber tile, and sand [12]. This study showed only minimal force reduction with the various surface materials, thus explaining the lack of convincing evidence from clinical studies on the effectiveness of playground surface materials in preventing distal radial fractures.

The strengths of our study include the use of chart review for data collection rather than utilizing databases [2-3]. Thus there was no concern for incorrect coding resulting in missed or incorrectly included cases. Given that no upper extremity injuries were missed by evaluating the convenience sample, we feel confident in our case finding technique for such injuries. This was also one of the few studies that visited the physical site of the injury to take measurements rather than utilizing a phone survey [7].

Limitations of our study include problems inherent in a retrospective analysis. There was incomplete data which made it difficult to know how many of the playground injuries occurred on monkey bars. Additionally, as many of the families contacted did not provide us with the location of the monkey bars, all injury-related equipment was not measured. Finally, as we were not able to determine the years the various monkeys were constructed we are unable to know if the monkey bars were in compliance with the recommendations of the years they were constructed.

It is worth noting that in 1996, there were 83,400 trampoline-related injuries in the United States (very similar to the 79,023 monkey bar injuries in 2017) [1]. This prompted a statement in 1999 by the American Academy of Pediatrics (AAP) Committee on Injury and Poison Prevention and Committee on Sports Medicine and Fitness to reaffirm its recommendation that trampolines should never be used in the home environment, in routine physical education classes or in outdoor playgrounds [13]. Such professional society advocacy statements and campaigns have resulted in a decline in trampoline injuries [14].

We believe the AAP and other societies need to address the ongoing concerns of serious injury related to monkey bars. There should be a strong consideration to either recommend against their use in playgrounds and at home or decrease the maximal height of the monkey bars to 150 cm or lower for all ages as suggested by the existing data [8-9]. Also, consider strategies to improve compliance with the recommendations.

## Conclusions

Monkey bars continue to result in large numbers of significant injuries, conservatively accounting for 6.7% of all upper extremity injuries and 3.4% of all pediatric injuries seen within the orthopedic group in 2017. Non-compliance with the US safety standards is common, such that 36.7% exceeded the current recommended maximal height for school-age children, and 100% exceed the recommended height for preschool children.
